# Food Is Reservoir of MDR *Salmonella*: Prevalence of ESBLs Profiles and Resistance Genes in Strains Isolated from Food

**DOI:** 10.3390/microorganisms10040780

**Published:** 2022-04-06

**Authors:** Delia Gambino, Valeria Gargano, Gaspare Butera, Sonia Sciortino, Mariangela Pizzo, Giuseppa Oliveri, Cinzia Cardamone, Chiara Piraino, Giovanni Cassata, Domenico Vicari, Antonella Costa

**Affiliations:** Istituto Zooprofilattico Sperimentale della Sicilia “A. Mirri”, 90129 Palermo, Italy; deliagamb@gmail.com (D.G.); gaspare.b@alice.it (G.B.); sonia.sciortino77@gmail.com (S.S.); mariangela.pizzo22@gmail.com (M.P.); giuseppa.oliveri@izssicilia.it (G.O.); cinzia.cardamone@izssicilia.it (C.C.); chiara.piraino@izssicilia.it (C.P.); giovanni.cassata@izssicilia.it (G.C.); domenico.vicari@izssicilia.it (D.V.); antonella.costa@izssicilia.it (A.C.)

**Keywords:** *Salmonella*, food pathogens, *S. Infantis*, antibiotic resistance, resistance gene, ESBLs

## Abstract

*Salmonella* spp. are among the most frequent causes of foodborne diseases, and the increasing occurrence of MDR strains is an additional cause for concern. In the three-year period 2019–2021, we collected *Salmonella* spp. strains isolated from different food categories analysed in the context of Regulation (EC) No 2073/2005 in order to assess their antibiotic susceptibility profiles and ESBL production. To determine the susceptibility profiles and identify MDR strains, we used the Kirby–Bauer method to test 17 antibiotics. Double-disc and PCR testing then allowed us to assess the production of ESBLs and the presence of beta-lactamase resistance genes. Phenotypic tests showed that 36 out of 67 strains were MDR and 52.7% of these were ESBL producers. Finally, molecular investigations conducted on ESBL-producing strains revealed the presence of *bla*_SHV_, *bla*_CTX-M_ and *bla*_TEM_ genes. Our results confirmed the prevalence of *S. Infantis*, an MDR strain and ESBL producer, in chicken meat. This suggests that further research on the prevalence of antibiotic resistance genes (ARGs) in foodborne strains is needed, especially from a One Health perspective.

## 1. Introduction

Salmonellosis is a commonly reported gastrointestinal infection in humans, and an important cause of foodborne outbreaks. In the European Union (EU) in 2019, the number of confirmed salmonellosis cases was 87,923; in 2020, the number was 57,702, which was the lowest recorded number since 2007 because of the impacts of the withdrawal of the United Kingdom from the EU and the COVID-19 pandemic [1]. The main route of infection is ingestion of food or water contaminated with *Salmonella* spp., Gram-negative, facultative anaerobic bacilli belonging to the *Enterobacteriaceae* family [2,3]. *Salmonella* is ubiquitous in the human food chain and is one of the most important foodborne pathogens in the world. In particular, *S. Enteritidis*, *S. Typhimurium*, monophasic *S. Typhimurium*, *S. Infantis* and *S. Derby* are the five serotypes most commonly involved in human infections [1]. In the EU, microbiological food controls carried out in the context of Regulation (EC) No. 2073/2005 found the highest percentages of *Salmonella*-positive samples in egg products, poultry meat and poultry products, which are the most critical sources of *Salmonella* spp. transmission to humans [1,4].

Although salmonellosis is generally self-limited and usually does not require specific treatment, antibiotic therapy with quinolones, beta-lactams, aminoglycosides, tetracyclines or sulfamethoxazole–trimethoprim is necessary in severe cases [5]. However, the overuse of antibiotics has contributed to the selection of MDR *Salmonella* strains, i.e., resistant simultaneously to three or more classes of antibiotics, including those most commonly prescribed for the treatment of salmonellosis [6]. The spread of MDR *Salmonella* represents a significant health problem, as it causes longer hospitalisations, prolonged illnesses and higher mortality rates than susceptible strains [7,8]. The World Health Organization estimates that of the 100,000 cases of salmonellosis each year, a large number are caused by MDR *Salmonella* [9], with the majority acquired through the consumption of contaminated food of animal origin, particularly beef, pork and poultry products [10,11].

In *Enterobacteriaceae* such as *Salmonella*, the main mechanism of resistance to beta-lactams is the acquisition of genes (*bla* gene) that encode for beta-lactamase hydrolytic enzymes, which inactivate the antibiotic [12]. Extended-spectrum beta-lactamases (ESBLs), which hydrolyse first-, second-, and third-generation penicillins and cephalosporins, are encoded by genes belonging to the TEM, SHV, and CTX-M families, including multiple variants of the *bla*_TEM_, *bla*_SHV_ and *bla*_CTX-M_ genes [13]. These ESBL genes have been identified in bacteria isolated from animals and food products of animal origin [7,14], as well as from other types of foods, such as seafood [15], raw vegetables [16] and ready-to-eat (RTE) foods [17], suggesting the possible role of the food production chain as a reservoir for this group of bacteria. Indeed, factors such as selective pressure in animal and environmental microbiomes, the circulation of bacteria between animals and environment and ineffective food safety management can contribute to the presence and persistence of antibiotic-resistant bacteria (ARB) and antibiotic resistance genes (ARGs) in the food production context [18].

The aim of this study was to evaluate the MDR potential of *Salmonella* strains isolated in the period from January 2019 to December 2021 from food samples analysed in the context of the Regulation (EC) No 2073/2005 [4]. Furthermore, for every MDR *Salmonella* strain, ESBL production and ESBL gene presence were determined by double-disc diffusion and PCR tests, respectively.

## 2. Materials and Methods

### 2.1. Salmonella Isolation

From January 2019 to December 2021, 493 food samples, subjected to controls according to European Community legislation, were analysed [4]. Specifically, these samples were poultry meat (*n* = 145), pig meat (*n* = 106), beef (*n* = 54), bivalve molluscs (*n* = 109), eggs (*n* = 43) and sprouted seeds (*n* = 36).

Isolation according to ISO 6579-1:2017 was performed, and strains were then identified by biochemical enzymatic assays and serotyping, according to the Kauffmann–White–Le Minor scheme (Supplementary Materials Table S1) [19].

### 2.2. Antibiotic Susceptibility Profile Determination

Antibiotic susceptibility was assessed using the Kirby–Bauer method on Mueller Hinton agar medium (Oxoid, Milan, Italy), testing 17 antibiotics: kanamycin (30 µg), gentamicin (10 µg), streptomycin (10 µg), tobramycin (10 µg), ampicillin (10 µg), amoxicillin/clavulanic acid (30 µg), cefotaxime (30 µg), ceftriaxone (30 µg), ceftazidime (30 µg), imipenem (10 µg), nalidixic acid (30 µg), ciprofloxacin (5 µg), enrofloxacin (5 µg), levofloxacin (5 µg), sulfamethoxazole/trimethoprim (25 µg), tetracycline (30 µg) and chloramphenicol (30 µg).

Interpretation of inhibition zones and classification of isolates as susceptible (S), intermediate (I) or resistant (R), was done in accordance with CLSI guidelines [20].

### 2.3. ESBL Production Evaluation by Double-Disc Test

The double-disc test (DDT) was conducted on 36 MDR *Salmonella* strains to phenotypically assess ESBL production. Discs containing cephalosporins (cefotaxime 30 µg, ceftazidime 30 µg, cefepime 30 µg) were placed next to a disc with clavulanic acid (30 µg amoxicillin–clavulanic acid), as recommended by EUCAST [21]. When zones of inhibition around any of the cephalosporin discs were increased or there was a ‘keyhole’ in the direction of amoxicillin–clavulanic acid disc, the test was considered positive.

### 2.4. Detection of Beta-Lactamase Genes

The beta-lactamase gene detection was conducted on the 19 strains that were found by the double-disc test to be ESBL-producing. Bacterial DNA was extracted using 100 µL of PrepMan™ ultra Sample Preparation Reagent (Thermo Fisher Scientific, Waltham, MA, USA), according to the procedure recommended by the manufacturer. Real Time PCR reactions were performed using 10 ng of DNA template and 0.5 µM of the forward and reverse primers listed in Table 1, for a total volume of 25 µL of 1X of Advanced Universal SYBR Green Supermix (Bio-Rad Laboratories, Hercules, CA, USA), in order to amplify *bla*_TEM_, *bla*_CTX-M_, *bla*_SHV_ and *bla*_OXA_ genes.

The amplification program included an initial denaturation at 94 °C for 10 min, followed by 32 cycles of 94 °C for 30 s, 60 °C for 30 s, 72 °C for 15 s, and a final extension at 72 °C for 10 min. Subsequently, 10 µL of the PCR product were used for electrophoresis on 2% E-Gel™ Go! Agarose Gels (Thermo Fisher Scientific, Waltham, MA, USA) to determine the size of the product. In each Real Time PCR reaction, a positive and a negative control were used. The positive one was represented by DNA belonging to a strain of *Salmonella* in which the presence of the *bla* gene was previous confirmed by sequencing; the negative control was represented by a Not Template Control (NTC), in which the reaction volume with DNase free water was obtained.

## 3. Results

### 3.1. Isolation Results

**Figure 1:**
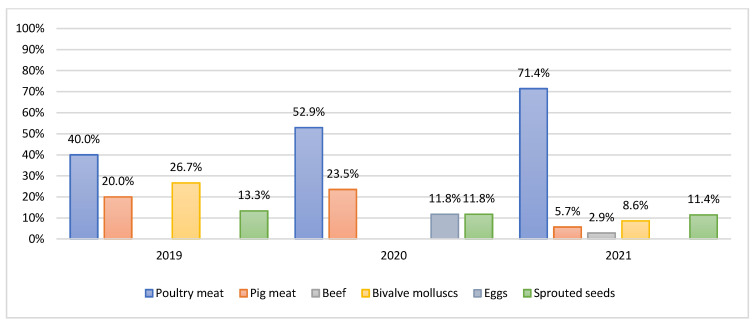
Prevalence per year of *Salmonella* based on food.

Microbiological analysis of the 493 food samples resulted in the isolation of 67 strains of *Salmonella* spp. (15 out of 172 were isolated in 2019, 17 out of 132 in 2020 and 35 out of 189 in 2021). Supplementary Materials Table S1 shows the samples that tested positive for the presence of *Salmonella* spp. and the serotypes identified. Notably, poultry meat was the main source of *Salmonella*, showing a prevalence of 40%, 52.9% and 71.4% in 2019, 2020 and 2021, respectively (Figure 1).

**Figure 2:**
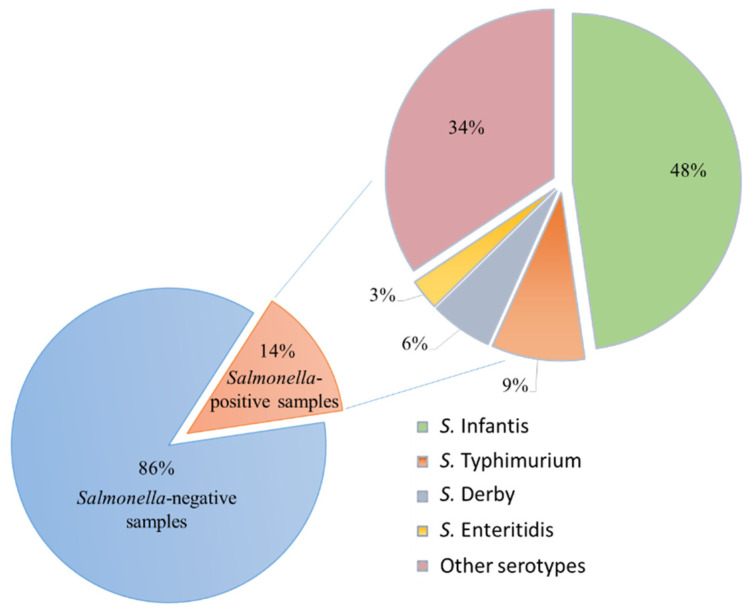
*Salmonella* spp. research results and serotypes identified in the 2019–2021 three-year period.

*S*. *Infantis* was the predominant serotype (48%), present in 32 poultry meat samples. Instead, *S.* *Typhimurium* (9%), *S. Derby* (6%) and *S. Enteritidis* (3%) serotypes were found to have a low prevalence (Figure 2).

### 3.2. Antibiotic Susceptibility and ESBL Production Test Results

Antibiotic susceptibility testing conducted on the 67 *Salmonella* strains showed the absence of resistance in 24 of these strains, whereas 43 strains (64%) were resistant to one or more of the tested antibiotics. Supplementary Materials Table S1 provides an overview of these strains and their resistances.

Notably, 31.3% of these strains were resistant to kanamycin, 43.2% to sulphonamides, 47.7% to nalidixic acid, 49.2% to ampicillin and 50.7% to tetracycline. Few strains showed resistance to levofloxacin (5%) or chloramphenicol (6%), whereas no resistance against imipenem, ciprofloxacin or enrofloxacin was detected.

An MDR profile was found in 36 strains that showed resistance to three (*n* = 4), four (*n* = 22) and five (*n* = 10) antibiotic classes (Supplementary Materials Table S1). Specifically, the most frequent MDR profiles were: aminoglycosides, beta-lactams, quinolones, sulphonamides and tetracyclines; resistance to these was found in eight *S. Infantis*, one *S. Salamae* and one *S. Kentucky*. Resistance to beta-lactams, quinolones, sulphonamides and tetracyclines was found in nine *S. Infantis* and one *S. Cerro*.

Finally, the double-disc test allowed detection of ESBL production in 19 strains. Indeed, for these strains, an increase in the zones of inhibition in the direction of amoxicillin or clavulanic acid was recorded around the tested cephalosporins (Table 2).

### 3.3. Detection of Beta-Lactamase Genes

Genes responsible for beta-lactamase activity in 19 ESBL-producing *Salmonella* strains were screened by PCR. The presence of beta-lactamase genes was detected in all tested strains, confirming the phenotypic results of ESBL production tests (Table 3).

The most frequently identified genes were *bla*_SHV_ and *bla*_CTX-M_, which were present in 68.4% and 47.3% of strains, respectively. Furthermore, the *bla*_TEM_ gene was harboured by only one strain, while *bla*_OXA_ was not detected. Specifically, nine strains harboured only the *bla*_SHV_ gene, six strains harboured only the *bla*_CTX-M_ gene, three strains harboured the *bla*_CTX-M_ and *bla*_SHV_ genes together, and one strain harboured the *bla*_TEM_ and *bla*_SHV_ genes together.

## 4. Discussion

*Salmonella* spp. are among the most frequent causes of foodborne diseases, and the increasing occurrence of MDR strains is an additional cause for concern. Thus, in the three-year period 2019–2021, we collected *Salmonella* spp. strains isolated from different food categories analysed in the context of Regulation (EC) No 2073/2005 [4], in order to assess their antibiotic susceptibility profiles and ESBL production.

Our data show that among the different food categories analysed, poultry meat was a relevant source of *Salmonella*. Moreover, regarding poultry meat, it is possible to note that the prevalence of *Salmonella* significantly increased over the three-year period, rising from 40% in 2019 to 71.4% in 2021; the prevalent serovar was *S. Infantis* (48%).

We performed a screening test using the Kirby–Bauer method to estimate the antibiotic susceptibility profiles of these strains, and we found a very high rate of strains showing at least one phenotypic resistance (64%). Among these, the highest rates of resistance were found against sulphonamides (43.2%), a class of antibiotics used in severe *Salmonella* infections, but also against nalidixic acid (47.7%) and kanamycin (31.3%). In addition, a high percentage of strains showed resistance to tetracyclines (50.7%), despite the fact that, in 2006, the European Union, in an attempt to counteract this trend, imposed a ban on the non-therapeutic use of antibiotics of human importance, such as tetracyclines, in farm animal feed. However, resistance to these drugs in *Salmonella* from food samples continues to be of concern [8,23]. This observation may be related to the human manipulation of these kinds of foods [24].

Of the strains tested, 53.7% showed an MDR profile with resistance to four or five classes in the majority of strains. These data are alarming, not only because of the real risk for consumers of becoming infected with an MDR strain, but also because many of these strains showed resistance to antibiotic classes important in human medicine, such as beta-lactamases. Thus, in order to obtain a complete overview of the resistance profiles of all the MDR strains isolated, we conducted a double-disc test (DDT) for ESBL phenotype detection. This test is one of the four different methods for confirming the ESBL phenotype recommended by EUCAST [21]. Despite the EFSA 2018/2019 report’s observation of resistance to third-generation cephalosporins at the overall low levels of 1.8% and 1.2% for cefotaxime and ceftazidime, respectively, for *Salmonella* spp., our experiment indicated that 52% of all MDR strains had an ESBL phenotype [8]. Finally, because these phenotypes could be conferred by several ARGs [25], the detection of beta-lactamase genes was performed in order to confirm phenotypic pattern. The PCRs we conducted allowed us to identify at least one gene encoding for β-lactamase enzymes in each strain that had an ESBL profile (Table 3). The *bla*_CTX-M_ gene was present in 9 out of 19 ESBL strains, and in three of these, it was in association with the *bla*_SHV_ gene, which was found to be the most prevalent gene among our isolates, because of its detection in 12 out of 19 ESBL strains. The *bla*_CTX-M_ genes encode for extended-spectrum β-lactamases (ESBLs) frequently identified in Gram-negative pathogens. These types of enzymes are active against cephalosporins and monobactams (but not cephamycins or carbapenems), and are currently of great epidemiological and clinical interest [26]. The *bla*_SHV_ gene has been identified mainly in *Enterobacteriaceae* causing nocosomal infections, but also in isolates from different contexts (human, animal and environment) [27,28]. Probably originating from a chromosomal penicillinase of *Klebsiella pneumoniae*, SHV β-lactamases currently comprise a large number of allelic variants, including extended-spectrum β-lactamases (ESBLs), non-ESBLs and several unclassified variants [29]. Our isolates showed an ESBL phenotype, so we have probably identified *bla*_SHV_ genes encoded for extended-spectrum β-lactamases.

These data are certainly alarming, since all of our strains came from food samples, particularly poultry, intended for human consumption. Indeed, although cooking these products may reduce the risk of foodborne disease, ARGs can resist high temperatures and, once ingested, can be transferred to the gut microbiota and confer resistance to other bacteria [30]. Therefore, our data are in line with the latest EFSA recommendations, which confirm how important it is in the monitoring and surveillance of antibiotic resistance (AMR) to assess the presence of ARGs in foodborne strains, especially in a One Health approach that recognises the circularity of human, animal and environmental health.

## Figures and Tables

**Table 1:** Primers used in this study.

Target	Primers	Sequence (5′-3′)	Amplicon Size (bp)	Reference
*bla*_TEM_	*bla*_TEM__F	ATTCTTGAAGACGAAAGGGC	661	[22]
	*bla*_TEM__R	ACGCTCAGTGGAACGAAAAC		
*bla*_CTX-M_	*bla*_CTX-M__F	CGCTTTGCGATGTGCAG	585	
	*bla*_CTX-M__R	ACCGCGATATCGTTGGT		
*bla*_SHV_	*bla*_SHV__F	CACTCAAGGATGTATTGTG	807	
	*bla*_SHV__R	TTAGCGTTGCCAGTGCTCG		
*bla*_OXA_	*bla*_OXA__F	ACACAATACATATCAACTTCGC	590	
	*bla*_OXA__R	AGTGTGTTTAGAATGGTGATC		

**Table 2:** Resistance and ESBL production test results of the 36 MDR *Salmonella* strains.

ID	Food	*Salmonella* Serotype	Isolation Year	Resistance	ESBL Production
AL-3	Poultry meat	*S. Infantis*	2019	AMP, CTX, NAL, SXT, TET	−
AL-11	Poultry meat	*S. Newport*	2019	KAN, AMP, SXT, TET	+
AL-14	Poultry meat	*S. Infantis*	2019	KAN, AMP, CTX, NAL, TET	−
AL-15	Poultry meat	*S. Infantis*	2019	KAN, AMP, CTX, NAL, SXT, TET	−
AL-30	Poultry meat	*S. Infantis*	2020	KAN, NAL, SXT, TET	+
AL-20	Poultry meat	*S. Infantis*	2020	NAL, SXT, TET	+
AL-21	Poultry meat	*S. Infantis*	2020	KAN, NAL, SXT, TET	+
AL-25	Poultry meat	*S. Infantis*	2020	STR, AMP, NAL, SXT, TET	+
AL-26	Poultry meat	*S. Infantis*	2020	STR, NAL, SXT, TET	+
AL-27	Poultry meat	*S. Infantis*	2020	KAN, STR, NAL, SXT, TET	+
AL-32	Poultry meat	*S. Infantis*	2020	KAN, STR, NAL, SXT, TET	+
AL-34	Poultry meat	*S. Infantis*	2021	KAN, STR, AMP, CTX, NAL, LEVO, CHL	−
AL-35	Poultry meat	*S. Infantis*	2021	KAN, STR, AMP, CTX, NAL, LEVO, CHL	−
AL-37	Poultry meat	*S. Agona*	2021	STR, AMP, SXT	+
AL-38	Pig meat	*S. Salamae*	2021	KAN, GEN, TOB, AMP, AMC, NAL, SXT, CHL	+
AL-39	Poultry meat	*S. Infantis*	2021	KAN, SXT, TET	+
AL-43	Poultry meat	*S. Infantis*	2021	KAN, AMP, STR, NAL, SXT, TET	+
AL-44	Poultry meat	*S. Infantis*	2021	KAN, TOB, AMP, CTX, CRO, NAL, SXT	−
AL-45	Poultry meat	*S. Infantis*	2021	AMP, CTX, CRO, NAL, SXT, TET	−
AL-46	Poultry meat	*S. Infantis*	2021	STR, AMP, NAL, TET	−
AL-47	Beef	*S. Cerro*	2021	AMP, AMC, CTX, CRO, NAL, SXT, TET	−
AL-48	Poultry meat	*S. Infantis*	2021	KAN, GEN, TOB, AMP, AMC, CTX, CRO, NAL, SXT, TET	−
AL-49	Poultry meat	*S. Infantis*	2021	KAN, AMP, AMC, CTX, CRO, NAL, SXT, TET	−
AL-50	Poultry meat	*S. Infantis*	2021	KAN, AMP, AMC, CTX, CRO, SXT, TET	−
AL-51	Pig meat	*S. Typhimurium*	2021	STR, AMP, TET	+
AL-52	Poultry meat	*S. Infantis*	2021	AMP, CTX, CAZ, CRO, NAL, SXT, TET	+
AL-53	Poultry meat	*S. Kentucky*	2021	STR, AMP, CAZ, CTX, CRO, NAL, SXT, TET	+
AL-56	Poultry meat	*S. Infantis*	2021	AMP, AMC, CRO, NAL, SXT, TET	−
AL-57	Poultry meat	*S. Infantis*	2021	TOB, AMP, AMC, CTX, CRO, NAL, SXT, TET	+
AL-58	Poultry meat	*S. Infantis*	2021	AMP, AMC, CTX, NAL, SXT, TET	+
AL-59	Poultry meat	*S. Infantis*	2021	GEN, AMP, CTX, NAL, SXT, TET	−
AL-60	Poultry meat	*S. Infantis*	2021	KAN, TOB, AMP, CTX, NAL, SXT, TET	+
AL-63	Poultry meat	*S. Infantis*	2021	STR, AMP, CTX, CAZ, NAL, SXT, TET, CHL	+
AL-65	Poultry meat	*S. Infantis*	2021	AMP, CTX, CAZ, CRO, NAL, SXT, TET	−
AL-66	Poultry meat	*S. Infantis*	2021	KAN, STR, AMP, NAL, TET	−
AL-67	Poultry meat	*S. Infantis*	2021	KAN, AMP, NAL, TET	−

AMP, Ampicillin; CTX, Cefotaxime; NAL, Nalidixic Acid; SXT, Sulphamethoxazole/Trimethoprim; TET, Tetracycline; KAN, Kanamycin; GEN, Gentamicin; STR, Streptomycin; TOB, Tobramycin; AMC, Amoxicillin/Clavulanic acid; CAZ, Ceftazidime; CRO, Ceftriaxone; LEVO, Levofloxacin; CHL, Chloramphenicol.

**Table 3:** Beta-lactamase resistance gene detection results.

ID Strains	Food	*Salmonella* Serotype	ESBL Production	*bla* Gene Detected
AL-11	Poultry meat	*S. Newport*	+	*bla*_TEM_, *bla*_SHV_
AL-30	Poultry meat	*S. Infantis*	+	*bla*_CTX-M_
AL-20	Poultry meat	*S. Infantis*	+	*bla*_SHV_
AL-21	Poultry meat	*S. Infantis*	+	*bla*_CTX-M_
AL-25	Poultry meat	*S. Infantis*	+	*bla*_SHV_
AL-26	Poultry meat	*S. Infantis*	+	*bla*_SHV_
AL-27	Poultry meat	*S. Infantis*	+	*bla*_CTX-M_
AL-32	Poultry meat	*S. Infantis*	+	*bla*_SHV_
AL-37	Poultry meat	*S. Agona*	+	*bla*_SHV_
AL-38	Pig meat	*S. Salamae*	+	*bla*_CTX-M_, *bla*_SHV_
AL-39	Poultry meat	*S. Infantis*	+	*bla*_SHV_
AL-43	Poultry meat	*S. Infantis*	+	*bla*_CTX-M_
AL-51	Pig meat	*S. Typhimurium*	+	*bla*_SHV_
AL-52	Poultry meat	*S. Infantis*	+	*bla*_SHV_
AL-53	Poultry meat	*S. Kentucky*	+	*bla*_CTX-M_
AL-57	Poultry meat	*S. Infantis*	+	*bla*_CTX-M_
AL-58	Poultry meat	*S. Infantis*	+	*bla*_CTX-M_, *bla*_SHV_
AL-60	Poultry meat	*S. Infantis*	+	*bla*_SHV_
AL-63	Poultry meat	*S. Infantis*	+	*bla*_CTX-M_, *bla*_SHV_

## Data Availability

All data discussed are contained in the manuscript.

## Supplementary Materials

The following supporting information can be downloaded at: https://www.mdpi.com/article/10.3390/microorganisms10040780/s1. Table S1: Analysed strains and their phenotypic resistances.
