# Supplementary material for: Food Is Reservoir of MDR Salmonella: Prevalence of ESBLs Profiles and Resistance Genes in Strains Isolated from Food
Source: Microorganisms. 2022 Apr 6;10(4):780. doi: 10.3390/microorganisms10040780 (PMC9026803; doi:10.3390/microorganisms10040780)
Supplement: Supplementary file 1 [file microorganisms-10-00780-s001.zip › microorganisms-1670452-supplementary.pdf]

Supplementary Table S1. Analysed serotypes and phenotypic resistances

Id. Strains	Province of Sample Origin (Sicily, Italy)	Food	Salmonella serotype	Isolation year	Resistance	
					Antibiotic	Antibiotic Classes (number)
AL-1	Palermo	Poultry meat	*S.* Typhimurium	2019	ND[1]	ND[1]
AL-2	Palermo	Bivalve molluscs	*S.* Derby	2019	AMP, TET	Beta-lactams, tetracyclines (2)
AL-3	Palermo	Poultry meat	*S.* Infantis	2019	AMP, CTX, NAL, SXT, TET	Beta-lactams, quinolones, sulfonamides, tetracyclines (4)
AL-4	Palermo	Pig meat	*S.* Derby	2019	ND[1]	ND[1]
AL-5	Palermo	Pig meat	*S.* Typhimurium	2019	ND[1]	ND[1]
AL-6	Palermo	Bivalve molluscs	*S.* Bredeney	2019	ND[1]	ND[1]
AL-7	Palermo	Bivalve molluscs	*S.* Bredeney	2019	ND[1]	ND[1]
AL-8	Palermo	Pig meat	*S.* Derby	2019	ND[1]	ND[1]
AL-9	Palermo	Poultry meat	*S.* Infantis	2019	TET	Tetracyclines (1)
AL-10	Agrigento	Sprouted seeds	*S.* Typhimurium	2019	ND[1]	ND[1]
AL-11	Palermo	Poultry meat	*S.* Newport	2019	KAN, AMP, SXT, TET	Aminoglycosides, beta-lactams, sulfonamides, tetracyclines (4)
AL-12	Palermo	Bivalve molluscs	*S.* Cardoner	2019	ND[1]	ND[1]
AL-13	Palermo	Sprouted seeds	*S. bongori*	2019	ND[1]	ND[1]
AL-14	Palermo	Poultry meat	*S.* Infantis	2019	KAN, AMP, CTX, NAL, TET	Aminoglycosides, beta-lactams, quinolones, tetracyclines (4)
AL-15	Trapani	Poultry meat	*S.* Infantis	2019	KAN, AMP, CTX, NAL, SXT, TET	Beta-lactams, quinolones, sulfonamides, tetracyclines (4)
AL-29	Palermo	Pig meat	*S.* Derby	2020	ND[1]	ND[1]
AL-30	Palermo	Poultry meat	*S.* Infantis	2020	KAN, NAL, SXT, TET	Beta-lactams, quinolones, sulfonamides, tetracyclines (4)
AL-16	Palermo	Pig meat	*S.* Typhimurium monophasic var.	2020	TET	Tetracyclines (1)
AL-17	Trapani	Poultry meat	*S.* Kentucky	2020	ND[1]	ND[1]
AL-18	Trapani	Poultry meat	*S.* Kentucky	2020	ND[1]	ND[1]
AL-19	Trapani	Eggs	*S.* Enteritidis	2020	ND[1]	ND[1]
AL-20	Trapani	Poultry meat	*S.* Infantis	2020	NAL, SXT, TET	Quinolones, sulfonamides, tetracyclines (3)
AL-21	Agrigento	Poultry meat	*S.* Infantis	2020	KAN, NAL, SXT, TET	Aminoglycosides, quinolones, sulfonamides, tetracyclines (4)
AL-22	Palermo	Pig meat	*S.* Kedougou	2020	ND[1]	ND[1]
AL-23	Palermo	Sprouted seeds	*S.* Rhydyfelin	2020	ND[1]	ND[1]
AL-24	Palermo	Pig meat	*S.* Derby	2020	ND[1]	ND[1]

AL-25	Palermo	Poultry meat	*S.* Infantis	2020	STR, AMP, NAL, SXT, TET	Aminoglycosides, beta-lactams, quinolones, sulfonamides, tetracyclines (5)
AL-26	Trapani	Poultry meat	*S.* Infantis	2020	STR, NAL, SXT, TET	Aminoglycosides, quinolones, sulfonamides, tetracyclines (4)
AL-27	Trapani	Poultry meat	*S.* Infantis	2020	KAN, STR, NAL, SXT, TET	Aminoglycosides, quinolones, sulfonamides, tetracyclines (4)
AL-28	Palermo	Eggs	*S.* Enteritidis	2020	ND[1]	ND[1]
AL-31	Palermo	Sprouted seeds	*S.* Isangi	2020	ND[1]	ND[1]
AL-32	Trapani	Poultry meat	*S.* Infantis	2020	KAN, STR, NAL, SXT, TET	Aminoglycosides, quinolones, sulfonamides, tetracyclines (4)
AL-33	Agrigento	Sprouted seeds	*S. bongori*	2021	ND[1]	ND[1]
AL-34	Agrigento	Poultry meat	*S.* Infantis	2021	KAN, STR, AMP, CTX, NAL, LEV, CHL	Aminoglycosides, beta-lactams, quinolones, phenicoles (4)
AL-35	Agrigento	Poultry meat	*S.* Infantis	2021	KAN, STR, AMP, CTX, NAL, LEV, CHL	Aminoglycosides, beta-lactams, quinolones, phenicoles (4)
AL-36	Palermo	Poultry meat	*S.* Agona	2021	ND[1]	ND[1]
AL-37	Trapani	Poultry meat	*S.* Agona	2021	STR, AMP, SXT	Aminoglycosides, beta-lactams, sulfonamides (3)
AL-38	Palermo	Pig meat	*S.* Salamae	2021	KAN, GEN, TOB, AMP, AMC, NAL, SXT, CHL	Aminoglycosides, beta-lactams, quinolones, sulfonamides, tetracyclines (5)
AL-39	Palermo	Poultry meat	*S.* Infantis	2021	KAN, SXT, TET	Quinolones, sulfonamides, tetracyclines (3)
AL-40	Palermo	Sprouted seeds	*S.* Kundachi	2021	ND[1]	ND[1]
AL-41	Palermo	Sprouted seeds	*S.* Chandas	2021	ND[1]	ND[1]
AL-42	Palermo	Sprouted seeds	*S.* Colobane	2021	ND[1]	ND[1]
AL-43	Agrigento	Poultry meat	*S.* Infantis	2021	KAN, AMP, STR, NAL, SXT, TET	Aminoglycosides, beta-lactams, quinolones, sulfonamides, tetracyclines (5)
AL-44	Agrigento	Poultry meat	*S.* Infantis	2021	KAN, TOB, AMP, CTX, CRO, NAL, SXT	Aminoglycosides, beta-lactams, quinolones, sulfonamides (4)
AL-45	Agrigento	Poultry meat	*S.* Infantis	2021	AMP, CTX, CRO, NAL, SXT, TET	Beta-lactams, quinolones, sulfonamides, tetracyclines (4)
AL-46	Palermo	Poultry meat	*S.* Infantis	2021	STR, AMP, NAL, TET	Aminoglycosides, beta-lactams, quinolones , tetracyclines (4)
AL-47	Palermo	Beef	*S.* Cerro	2021	AMP, AMC, CTX, CRO, NAL, SXT, TET	Beta-lactams, quinolones, sulfonamides, tetracyclines (4)
AL-48	Palermo	Poultry meat	*S.* Infantis	2021	KAN, GEN, TOB, AMP, AMC, CTX, CRO, NAL, SXT, TET	Aminoglycosides, beta-lactams, quinolones, sulfonamides, tetracyclines (5)
AL-49	Palermo	Poultry meat	*S.* Infantis	2021	KAN, AMP, AMC, CTX, CRO, NAL, SXT, TET	Beta-lactams, quinolones, sulfonamides, tetracyclines (4)
AL-50	Palermo	Poultry meat	*S.* Infantis	2021	KAN, AMP, AMC, CTX, CRO, SXT, TET	Aminoglycosides, beta-lactams, quinolones, sulfonamides, tetracyclines (5)
AL-51	Trapani	Pig meat	*S.* Typhimurium	2021	STR, AMP, TET	Aminoglycosides, beta-lactams, tetracyclines (3)
AL-52	Agrigento	Poultry meat	*S.* Infantis	2021	AMP, CTX, CAZ, CRO, NAL, SXT, TET	Beta-lactams, quinolones, sulfonamides, tetracyclines (4)
AL-53	Agrigento	Poultry meat	*S.* Kentucky	2021	STR, AMP, CAZ, CTX, CRO, NAL, SXT, TET	Aminoglycosides, beta-lactams, quinolones, sulfonamides, tetracyclines (5)
AL-54	Agrigento	Poultry meat	*S.* Kentucky	2021	ND[1]	ND[1]

AL-55	Agrigento	Poultry meat	*S.* Infantis	2021	AMP	Beta-lactams (1)
AL-56	Ragusa	Poultry meat	*S.* Infantis	2021	AMP, AMC, CRO, NAL, SXT, TET	Beta lactams, quinolones, sulfonamides, tetracyclines (4)
AL-57	Ragusa	Poultry meat	*S.* Infantis	2021	TOB, AMP, AMC, CTX, CRO, NAL, SXT, TET	Aminoglycosides, beta-lactams, quinolones, sulfonamides, tetracyclines (5)
AL-58	Ragusa	Poultry meat	*S.* Infantis	2021	AMP, AMC, CTX, NAL, SXT, TET	Beta lactams, quinolones, sulfonamides, tetracyclines (4)
AL-59	Ragusa	Poultry meat	*S.* Infantis	2021	GEN, AMP, CTX, NAL, SXT, TET	Aminoglycosides, beta-lactams, quinolones, sulfonamides, tetracyclines (5)
AL-60	Ragusa	Poultry meat	*S.* Infantis	2021	KAN, TOB, AMP, CTX, NAL, SXT, TET	Aminoglycosides, beta-lactams, quinolones, sulfonamides, tetracyclines (5)
AL-61	Palermo	Bivalve molluscs	*S.* Corvallis	2021	KAN, GEN, TOB, AMP	Aminoglycosides, beta-lactams (2)
AL-62	Palermo	Bivalve molluscs	*S.* Corvallis	2021	KAN, GEN, TOB, AMP	Aminoglycosides, beta-lactams (2)
AL-63	Palermo	Poultry meat	*S.* Infantis	2021	STR, AMP, CTX, CAZ, NAL, SXT, TET, CHL	Aminoglycosides, beta-lactams, quinolones, sulfonamides, tetracyclines (5)
AL-64	Ragusa	Bivalve molluscs	*S.* Saintpaul	2021	NAL	Quinolones (1)
AL-65	Caltanissetta	Poultry meat	*S.* Infantis	2021	AMP, CTX, CAZ, CRO, NAL, SXT, TET	Beta lactams, quinolones, sulfonamides, tetracyclines (4)
AL-66	Caltanissetta	Poultry meat	*S.* Infantis	2021	KAN, STR, AMP, NAL, TET	Aminoglycosides, beta-lactams, quinolones , tetracyclines (4)
AL-67	Caltanissetta	Poultry meat	*S.* Infantis	2021	KAN, AMP, NAL, TET	Aminoglycosides, beta-lactams, quinolones , tetracyclines (4)

ND[1], not detected; AMP, Ampicillin; CTX, Cefotaxime; NAL, Nalidixic Acid; SXT, Sulphamethoxazole/Trimethoprim; TET, Tetracycline; Kanamycin, KAN; Gentamicin, GEN; Streptomycin, STR; Tobramycin, TOB; Amoxicillin/Clavulanic acid, AMC; Ceftazidime, CAZ; Ceftriaxone, CRO; Levofloxacin, LEVO; Chloramphenicol, CHL.
